# Note from the editors: Special issue on advanced diagnostics to inform public health policy

**DOI:** 10.2807/1560-7917.ES.2018.23.50.1812133

**Published:** 2018-12-13

**Authors:** Eurosurveillance editorial team

**Affiliations:** 1 European Centre for Disease Prevention and Control (ECDC), Stockholm, Sweden

This issue features the first three articles of the *Eurosurveillance* special issue on advanced diagnostics to inform public health policy. The second set of articles for the special issue will follow in mid-January.

Acknowledging the impact of advances in laboratory technology—including the application of qualitative and quantitative molecular diagnostics, proteomics (e.g. MALDI-TOF MS) and genomics, particularly whole genome sequencing (WGS) and metagenomics—on clinical and public health microbiology and consequently on public health, we issued a call for contributions for this special issue in late 2017 [1].

Here we present the first part of the special issue, with three articles that exemplify proof of concepts and novel applications of laboratory methods that have potential to impact public health and surveillance. In their study, van der Veer et al. applied *Neisseria gonorrhoeae* multi-antigen sequence typing (Ng-MAST) directly to urogenital and extra-genital samples, allowing for diagnosis of *N. gonorrhoeae* even in cases where culture failed, thus overcoming an important limitation of culture-dependent approaches [2]. Moreover, the authors were able to detect concurrent infections with distinct strains in the same patient, which would have been missed if they had followed the resistance surveillance guidelines that recommend culture of only one anatomical site.

An article on diagnostics of Legionnaires’ disease (LD) by researchers from Italy also demonstrates the advantages of a molecular, culture-independent method over the more time-intensive diagnostic approaches based on culture [3]. In this paper, Ricci et al. applied real-time PCR directly on respiratory specimens for the detection of LD and, based on their experiences, propose the adoption of PCR as routine laboratory testing to diagnose LD, as well as to consider the inclusion of PCR positivity in the case definition of a confirmed LD case.

In a proof of concept, Kafetzopoulou et al. demonstrate that metagenomic sequencing using Illumina MiSeq and the portable Oxford Nanopore MinION was able to recover whole genome sequences of chikungunya and dengue viruses directly from serum or plasma samples [4]. Authors inferred that the applied technique may also be used to detect other RNA viruses, thereby supporting front-line public health investigations during outbreak situations.

The research in these three articles provides novel ideas and opens new public health perspectives. We are confident that our readers will find them interesting and we look forward to presenting the second part of the special issue in mid-January 2019.
